# **The MOTION** **Study: A Randomized Controlled Trial with Objective Real-World Outcomes for Lumbar Spinal Stenosis Patients Treated with the *mild*^®^** Procedure**: One-Year Results**

**DOI:** 10.1093/pm/pnac028

**Published:** 2022-02-15

**Authors:** Timothy R Deer, Shrif J Costandi, Edward Washabaugh, Timothy B Chafin, Sayed E Wahezi, Navdeep Jassal, Dawood Sayed

**Affiliations:** 1 The Spine & Nerve Centers of the Virginias, Charleston, West Virginia; 2 Department of Pain Management, Cleveland Clinic, Cleveland, Ohio; 3 Michigan Pain Specialists, Ypsilanti, Michigan; 4 Vidant Roanoke-Chowan Hospital, Ahoskie, North Carolina; 5 Multidisciplinary Pain Program,Bronx, New York; 6 Spine & Pain Institute of Florida, Lakeland, Florida; 7 The University of Kansas Medical Center, Kansas City, Kansas, USA

**Keywords:** Lumbar Spinal Stenosis, *mild*, Minimally Invasive Lumbar Decompression, Ligamentum Flavum, Chronic Low Back Pain, Neurogenic Claudication

## Abstract

**Objective:**

The purpose of this study is to provide Level-1 objective, real-world outcome data for patients with lumbar spinal stenosis suffering from neurogenic claudication secondary to hypertrophic ligamentum flavum.

**Design:**

The MOTION Study is a prospective, multicenter, randomized controlled trial comparing the *mild*^®^ Procedure (minimally invasive lumbar decompression; Vertos Medical, Aliso Viejo, CA, USA) as a first-line therapy in combination with nonsurgical conventional medical management (CMM) vs CMM alone as the active control.

**Methods:**

Patients in the test group received the *mild* Procedure at baseline. Both the *mild*+CMM group and the control group were allowed unrestricted access to conventional real-world therapies. Patient-reported outcomes included the Oswestry Disability Index, the Zurich Claudication Questionnaire, and the Numeric Pain Rating Scale. A validated Walking Tolerance Test, the incidence of subsequent lumbar spine interventions, and the occurrence of adverse events were used to measure objective outcomes.

**Results:**

Sixty-nine patients in each group were analyzed at 1-year follow-up. No device- or procedure-related adverse events were reported in either group. Results from all primary and secondary outcome measures showed statistical significance in favor of *mild*+CMM.

**Conclusions:**

One-year results of this Level-1 study demonstrated superiority of *mild*+CMM over CMM alone for patients with lumbar spinal stenosis who were suffering from neurogenic claudication secondary to hypertrophic ligamentum flavum. Use of the validated Walking Tolerance Test to objectively measure increased ability to walk without severe symptoms provided evidence of statistically significantly better outcomes for *mild*+CMM than for CMM alone. With no reported device or procedure-related adverse events, the long-standing safety profile of the *mild* Procedure was reaffirmed. *mild* is a safe, durable, minimally invasive procedure that has been shown to be effective as an early interventional therapy for patients suffering from symptomatic lumbar spinal stenosis.

## Introduction

Lumbar spinal stenosis (LSS) is a degenerative spinal condition caused by narrowing of the spinal canal, which often results in compression of neural elements and symptoms of neurogenic claudication (NC) [1]. Patients with NC frequently present with multiple degenerative spinal conditions, including intervertebral disc bulging and herniation, facet arthropathy, and hypertrophic ligamentum flavum (HLF) [2]. HLF has been reported to contribute up to 85% of spinal canal narrowing [3]. The prevalence of LSS increases with age [4], and it can occur in one or more locations within the spinal canal (central, lateral, foraminal) [5].

Early treatment for patients with LSS generally begins with conservative measures, which can include physical therapy, home exercise programs, and oral analgesics, followed by low-risk interventional therapies [6–8]. First-line treatment may include the *mild*^®^ Procedure (minimally invasive lumbar decompression; Vertos Medical, Aliso Viejo, CA, USA) [9]. The *mild* Procedure provides minimally invasive decompression of the spinal canal for patients with LSS with NC secondary to HLF. The *mild* Procedure has demonstrated a better safety profile than have other spinal interventions, including surgical decompression, interspinous spacers, and fusion [9, 10]. Furthermore, the safety profile of *mild* has been shown to be similar to that of epidural steroid injections, with more durable results [10, 11].

The MOTION study compares *mild* in combination with conventional medical management (CMM) (*mild*+CMM) vs a CMM-only control group (CMM-Alone) in patients with LSS with NC. The study is designed to reflect real-world practice by allowing the use of standard-of-care CMM in both study groups at the discretion of the investigators. The only difference between the study groups is the use of *mild*, which is allowed in the *mild*+CMM group and not allowed in the CMM-Alone arm. In this study, the *mild* Procedure is used as first-line therapy together with other low-risk CMM treatment options. MOTION provides Level-1 evidence of the superiority of *mild*+CMM over CMM-Alone, as evaluated by multiple validated patient assessments and objective real-world outcome measures. Six-month results have been previously reported [12]. Primary endpoint results at 1-year follow-up are presented in the current report. Follow-up will continue annually through 5 years.

## Methods

### Study Design

The MOTION study is a prospective, multicenter, randomized controlled trial comparing the *mild* treatment in combination with CMM (*mild*+CMM) vs a CMM-only control group (CMM-Alone). The study is registered with the US Clinical Trial Registry as the MOTION Study (NCT03610737). It is being conducted at 19 interventional pain management centers throughout the United States. The study was approved by an Institutional Review Board for each participating site before patient enrollment, and it followed the Consolidated Standards of Reporting Trials (CONSORT) [13]. Records are maintained in compliance with the International Conference on Harmonization guidelines for Good Clinical Practices, and informed consent was obtained from all participants. This trial was registered on clinicaltrials.gov in August 2018, which was before enrollment of the first patient in September 2018.

### Patients

Patients ranging in age from 50 to 80 years and experiencing NC symptoms for a duration of at least 3 months were included in this study. Confirmation of patient eligibility involved an evaluation of medical history, including comorbidities, history of symptoms, and prior treatments, such as surgery and interspinous spacer. Baseline function was evaluated with the Oswestry Disability Index (ODI), Numerical Pain Rating Scale (NPRS), and Zurich Claudication Questionnaire (ZCQ) scores. Magnetic resonance images or computed tomographic images (when magnetic resonance imaging was not possible) of the spine were assessed by an independent medical monitor. MOTION study selection criteria are presented in Table 1.

**Table 1.** Eligibility criteria for the MOTION study

Inclusion Criteria	Exclusion Criteria
Age 50–80 years Patients experiencing NC symptoms for at least 3 months’ duration. LSS with NC diagnosed via: Symptomatic diagnosis [14] (see below)**and** Radiologic evidence of LSS with ligamentum flavum ≥2.5 mm [15] in thickness confirmed by preoperative magnetic resonance imaging or computed tomography performed within 12 months of baseline visit Stable opioid intake with no change during 30 days before enrollment. Available to complete all follow-up visits.	ODI score <31 (0–100 ODI Scale). NPRS score <5 (0–10 NPRS Scale). Lumbar epidural steroid injections during 8 weeks before study enrollment. Baseline analgesic medication greater than 90 milligram morphine equivalents per day. Prior surgery, interspinous spacer, intradiscal procedure, vertebral augmentation, or *mild* Procedure at the same treatment level. Radiofrequency ablation at the same or the adjacent levels within 6 months before study enrollment. History of spinal fractures with current related pain symptoms. Grade II or higher spondylolisthesis. Motor deficit or disabling back and/or leg pain from causes other than LSS NC. Unable to walk ≥10 feet unaided before being limited by pain. Previously randomized or treated in a similar clinical study. Epidural lipomatosis (if deemed to be a significant contributor of canal narrowing).
**NC Symptomatic Diagnosis**	
Pain/discomfort in leg, buttocks, or lower back while walking or standing. Bending forward or sitting down provides relief. Bending forward while walking. Unable to stand unaided without bending at the waist for more than 15 minutes. Unable to walk unaided without bending at the waist for more than one quarter mile.	

### Randomization

Patients with LSS were randomized in a 1:1 ratio to one of two parallel interventions for treatment of NC. In the test group, patients were treated with *mild*+CMM, whereas patients in the control group were treated with CMM alone. Study investigators at each site determined the appropriate CMM regimen for each patient. All primary and secondary endpoint outcomes were assessed at 1 year with comparisons to baseline. Crossover was allowed for patients in the CMM-Alone control group after 1-year follow-up. Subjects will continue study follow-up regardless of any crossover.

### Interventions

#### Conventional Medical Management

The appropriate CMM regimen for each MOTION patient was determined by the site investigator throughout the course of the study. As a reflection of real-world practice, CMM included any conservative or low-risk interventional therapies that are standard of care for early treatment of NC. CMM may be comprised of home exercise, walking aids, and physical therapy, as well as early interventional therapies, such as epidural steroid injections, medial branch injections, radiofrequency ablation, and facet joint injections.

#### The *mild* Procedure

The *mild* Procedure provides minimally invasive lumbar decompression performed from the posterior lumbar spinal approach with local anesthetic and light sedation. The procedure removes small portions of the lamina and preferentially resects and debulks the thickened ligamentum flavum, leaving no implants behind. The *mild* Procedure has been previously described in detail [9]. Patients assigned to the *mild*+CMM group underwent the *mild* Procedure after baseline assessment. The use of any additional CMM therapies was determined by the study investigator.

#### Outcome Measures

Multiple objective outcome measures were used to assess levels of function and pain for study patients. Objective outcome measures provide quality-of-recovery evaluations that are independent from judgment and are therefore less susceptible to bias [16–18]. These objective measures included a validated Walking Tolerance Test, in which patients were instructed to walk up to 15 minutes at their preferred speed, with the examination stopping at the end of 15 minutes or at the onset of severe symptoms, whichever came first [19].

Additional objective outcome measures included the number of subsequent lumbar spine interventions and safety data. Subsequent lumbar spine interventions that were defined to indicate study treatment nonresponse included laminectomy, laminotomy, lumbar fusion, interspinous spacers, neurostimulators, adhesiolysis, kyphoplasty, and additional *mild* Procedures. “Safety data” for this study refers to device- or procedure-related adverse events. All serious adverse events (SAEs) were reported, regardless of relationship. SAE was defined according to the CFR (US Code of Federal Regulations) Title 21 definition. All reportable adverse events were adjudicated by an independent clinical event adjudicator.

Subjective patient-reported outcomes included both primary and secondary patient-reported outcome measures. The primary efficacy endpoint was mean improvement in ODI score at 1-year follow-up compared with baseline. ODI is used to evaluate functional disability related to lower back pain [20]. Secondary endpoints included ZCQ and NPRS patient-reported outcomes. ZCQ assesses symptom severity and physical function specific to LSS, as well as patient satisfaction after treatment [21, 22]. NPRS measures the level of back and leg pain [23]. For each of these measures, patients receiving a subsequent lumbar spine intervention were considered nonresponders and represented a study failure with imputation of zero change from baseline.

#### Sample Size and Power

To test the primary superiority hypothesis, sample size was calculated to a threshold of 90% power. A sample size of 75 subjects in each group was considered sufficient under the assumption of a two-sided hypothesis with type 1 error of 0.05, power (1–β) of at least 90%, and randomization ratio of 1:1, with accounting for up to 10% data attrition. The randomized study population of N = 155 meets these criteria.

### Statistical Methods

Continuous data are summarized with means and standard deviations. Categorical variables are summarized with frequency counts and percentages. For multiple possible events occurring within a single patient (e.g., adverse events), the percentage is based on the number of patients experiencing the event. In this case, both patient and event counts are reported. Mean comparisons were performed with a two-tailed *t* test at a 0.05 level of significance and with a Fisher exact test for frequency in Microsoft Excel (Microsoft Corporation, Redmond, WA, USA) and VassarStats (Statistical Computation Website, http://www.vassarstats.net/).

## Results

### Patient Characteristics

**Figure 1.**
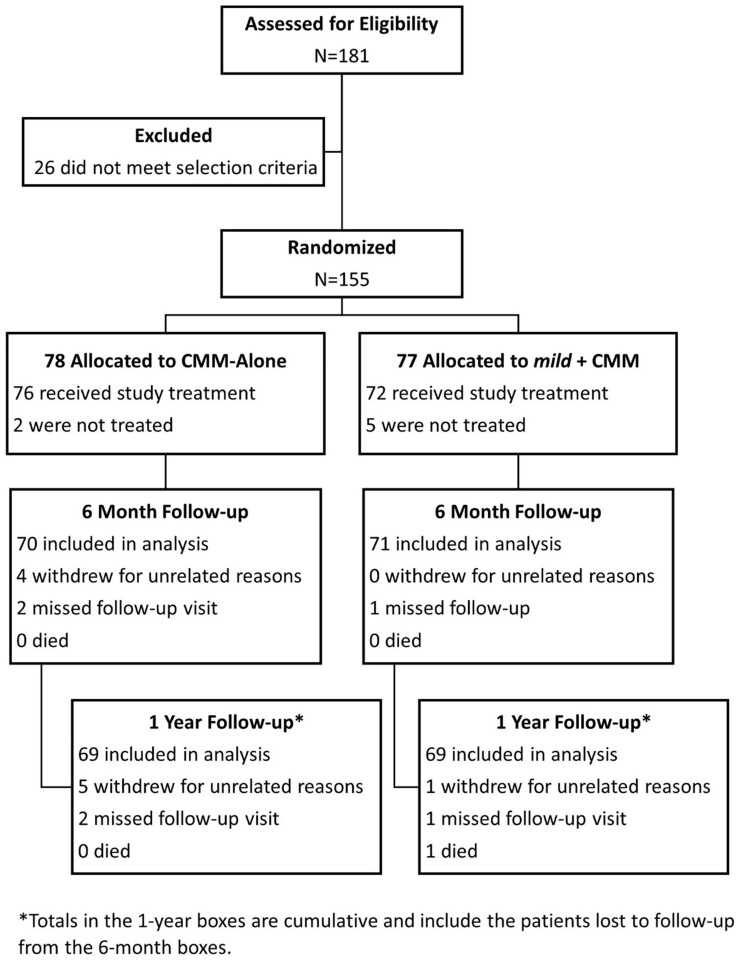
CONSORT diagram for patient flow through 1-year follow-up.

One hundred and eighty-one (n = 181) patients across 19 sites were evaluated for eligibility. Twenty-six (n = 26) did not meet the selection criteria and were excluded. The remaining 155 patients were randomized, with 77 assigned to the *mild*+CMM group and 78 to the CMM-Alone group. Five of 77 patients in the *mild*+CMM group and two of 78 patients in the CMM-Alone group were not treated. In the *mild*+CMM group, one patient withdrew for unrelated reasons, one missed a follow-up visit, and one died due to COVID-19. In the CMM-Alone group, five patients withdrew for unrelated reasons, and two missed follow-up visits. As a result, 69 patients were included in the analysis for each group at 1-year follow-up. See Figure 1 for detailed information in a CONSORT flow diagram.

**Figure 2.**
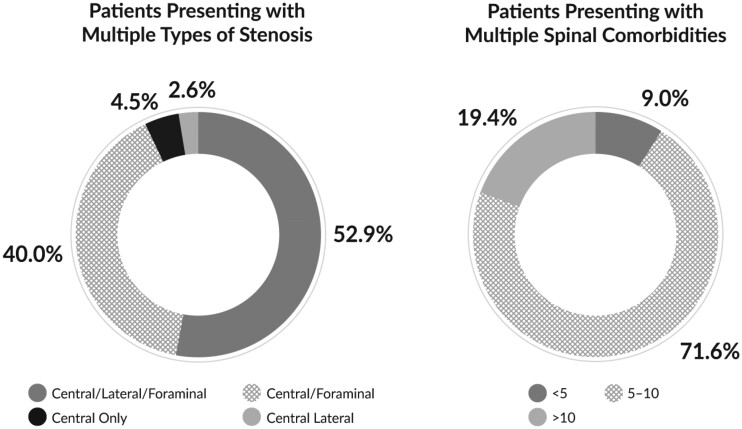
Percentage of MOTION patients presenting with multiple types of stenosis and spinal comorbidities. More than 90% of patients presented with foraminal as well as central stenosis, and the majority of patients suffered from all three types of stenosis: foraminal, lateral, and central. Ninety-one percent of patients presented with five or more spinal comorbidities.

At baseline, the average ages of *mild*+CMM and CMM-Alone patients were 64.7 years and 66.8 years, respectively. Patient age was not statistically different between the groups (*P* = 0.077). Other patient characteristics and baseline metrics are listed in Table 2. No difference in any of the demographic or baseline metrics was significant between the groups. A large majority of patients (95.5%) presented with multiple types of stenosis, and 91.0% presented with five or more spinal comorbidities (Figure 2). The most common spinal comorbidities were noted as HLF, foraminal narrowing, bulging disc, facet arthropathy, facet hypertrophy, degenerative disc disease, lateral recess narrowing, and disc space loss, with no significant differences in the rates of these comorbidities between the groups (*P* values ranging from 0.246 to 1.000). A complete list of spinal comorbidities was previously reported in the MOTION 6-month report [12].

**Table 2.** Patient demographics and baseline outcome metrics

	CMM-Alone (n = 78)	*mild*+CMM (n = 77)	*P* Value
Demographics			
Age, years	66.8 ± 7.5	64.7 ± 7.4	0.077
Gender, % (n)			1.000
Male	42.3% (33)	42.9% (33)	
Female	57.7% (45)	57.1% (44)	
Body mass index, kg/m^2^	32.0 ± 6.2	33.3 ± 7.8	0.347
Baseline metrics			
ODI	51.7 ± 14.8	55.3 ± 14.3	0.129
NPRS*	7.8 ± 1.5	7.5 ± 1.4	0.259
ZCQ Symptom Severity	3.56 ± 0.59	3.58 ± 0.61	0.887
ZCQ Physical Function	2.78 ± 0.46	2.84 ± 0.50	0.425

Values are given as mean ± standard deviation or % (n).

* A combined back + leg NPRS score was obtained at baseline, whereas separate back and leg scores were measured at follow-up. The combined baseline score was used in the calculations for NPRS score changes at follow-up.

### The mild Procedure

The majority of patients who received *mild* in the *mild*+CMM group were treated at one level only (59.7%), and L4–L5 was the most commonly treated level (76.4%), followed by L3–L4 (52.8%). Most patients were treated bilaterally (90.3%). Nearly two thirds of *mild* Procedures were performed in the hospital outpatient setting (65.3%), and the balance (34.7%) were conducted in an ambulatory surgery center. The large majority of *mild* patients received monitored anesthesia care sedation (93.1%), which includes light sedation and monitored anesthesia care with local anesthesia. Table 3 presents full details of the *mild* Procedure characteristics for patients treated in the *mild*+CMM group.

**Table 3.** Characteristics of the *mild* Procedure (n = 72)

Characteristic	% (n)
Procedure setting	
Ambulatory surgery center	34.7% (25)
Hospital outpatient	65.3% (47)
Anesthesia type	
Monitored anesthesia care (MAC)*	93.1% (67)
General^†^	4.2% (3)
Local only	2.8% (2)
Surgical approach	
Unilateral treatment	9.7% (7)
Bilateral treatment	90.3% (65)
Levels treated	
L2–L3	13.9% (10)
L3–L4	52.8% (38)
L4–L5	76.4% (55)
L5–S1	5.6% (4)
Number of levels treated	
1	59.7% (43)
2	31.9% (23)
3	8.3% (6)

* MAC includes light sedation and MAC with local anesthetic.

† General includes general anesthesia with local anesthetic.

All CMM therapies performed in this study are listed in Table 4. Some patients in both groups opted for no CMM therapy. Home exercise programs and walking regimens are often encouraged for patients undergoing *mild*, who generally have no activity restrictions within 24 hours of treatment and can immediately proceed with reconditioning. Overall, the CMM-Alone group received a higher rate of interventional therapies than did the *mild*+CMM group (61.3% vs 47.9%), but this difference was not statistically significant.

**Table 4.** CMM therapies received

Treatment	CMM-Alone (n = 62*)	*mild*+CMM (n = 71^†^)	*P* Value
Total—received CMM therapies, % (n)	83.9% (52)	76.1% (54)	0.288
Multiple CMM therapies, % (n)	64.5% (40)	54.9%(39)	0.292
Conservative therapy, % (n)			
Total conservative therapy	69.4% (43)	66.2% (47)	0.715
Home exercise	30.6% (19)	39.4% (28)	0.364
Walking aid	19.4% (12)	28.2% (20)	0.310
Physical therapy	38.7% (24)	23.9% (17)	0.090
Other^‡^	29.0% (18)	28.2% (20)	1.000
Interventional therapy, % (n)			
Total interventional therapy	61.3% (38)	47.9% (34)	0.163
Lumbar epidural steroid injections	51.6% (32)	38.0% (27)	0.161
Sacroiliac joint injection	8.1% (5)	11.3% (8)	0.574
Medial branch injection	12.9% (8)	5.6% (4)	0.225
Radiofrequency ablation	8.1% (5)	5.6% (4)	0.733
Other^‡^	21.0% (13)	15.5% (11)	0.500

* CMM data were not available for seven patients.

† Two patients had 6-month follow-up but missed 1-year follow-up.

‡“ Other” conservative therapy includes back brace, bed rest, aquatic therapy, acupuncture, chiropractic, transcutaneous electrical nerve stimulation (TENS), activity restriction, inversion table, heat, yoga, and massage. “Other” interventional therapy includes greater trochanteric bursa injection, hip/knee injections, platelet-rich plasma, nonlumbar epidural steroid injection, and facet and trigger point injections.

### Objective Outcome Measures

**Figure 3.**
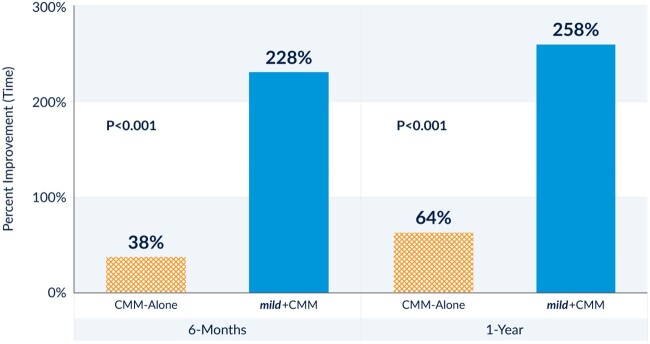
Walking Tolerance Test mean percent improvement at 6-month and 1-year follow-ups. At 1-year follow-up, *mild*+CMM patients achieved a mean improvement of 258% in walking time to onset of severe symptoms, compared with a 64% mean improvement for CMM-Alone patients. This difference between groups was statistically significant at both the 6-month and 1-year follow-ups (*P* < 0.001).

Objective outcomes were measured with real-world assessments, including improvement in walking, incidence of subsequent lumbar spine interventions, and safety. The Walking Tolerance Test demonstrated that patients in the *mild*+CMM group achieved a mean improvement of 258% in walking time to onset of severe symptoms, compared with a 64% mean improvement for patients in the CMM-Alone group. This difference between groups was statistically significant (*P* < 0.001) (Figure 3).

**Figure 4.**
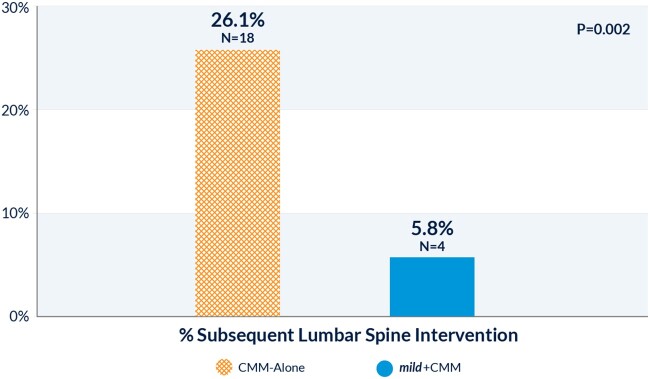
Incidence of subsequent lumbar spine interventions at 1-year follow-up. At 1-year follow-up, 26.1% of CMM-Alone patients had undergone a subsequent lumbar spine intervention, compared with 5.8% of *mild*+CMM patients (*P* = 0.002). This represents a 4.5-times higher rate of subsequent lumbar spine interventions for patients in the CMM-Alone group than for those in the *mild*+CMM group.

At 1-year follow-up, 26.1% of CMM-Alone patients had undergone a subsequent lumbar spine intervention, compared with 5.8% of *mild*+CMM patients (*P* = 0.002) (Figure 4). This represents a nearly five-fold higher rate of subsequent lumbar spine interventions for patients in the CMM-Alone group. Subsequent procedures in the CMM-Alone group included *mild* Procedures (n = 9), laminectomies (n = 2), fusion (n = 1), stimulator (n = 1), and spacer (n = 1). In the *mild*+CMM group, subsequent lumbar spine interventions were spacers (n = 2), surgical decompression (n = 1), and stimulator (n = 1).

There were no device- or procedure-related adverse events reported in either study group, and therefore safety was similar between the groups at 1 year. In the CMM-Alone group, seven patients experienced eight unrelated SAEs (11.5%), and in the *mild*+CMM group, eight patients experienced 13 unrelated SAEs (11.7%) (*P* = 0.793).

### Subjective Patient-Reported Outcome Measures

**Figure 5.**
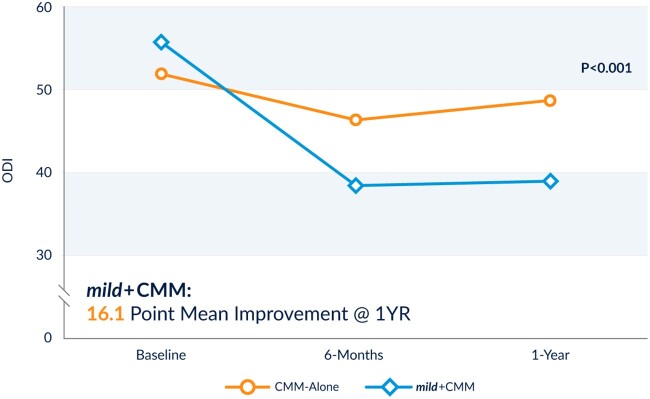
ODI outcomes at 6 months and 1 year. This comparison of ODI mean values over time shows statistically significantly better improvements in the *mild*+CMM arm than in the CMM-Alone group at both the 6-month and 1-year follow-ups.

At 1-year follow-up, patients in the *mild*+CMM group experienced a 16.1-point composite ODI mean improvement, compared with a 2.0-point mean improvement for patients in the CMM-Alone arm. This difference was statistically significant and indicates superiority of *mild*+CMM (*P* < 0.001) (Figure 5). See Table 5 for ODI mean change and additional data at the 6-month and 1-year follow-ups.

**Table 5.** Primary and secondary outcome 6-month and 1-year results

Outcome Measures Mean Improvement		6 Months			1 Year		
		CMM-Alone (n = 70)	*mild*+CMM (n = 71)	*P* Value*	CMM-Alone (n = 69)	*mild*+CMM (n = 69)	*P* Value*
**ODI**							
Mean ± SD		3.8 ± 11.1	16.3 ± 18.0	<0.001	2.0 ± 11.7	16.1 ± 19.0	<0.001
*P* value (within group)		0.005	<0.001	–	0.155^§^	<0.001	–
**NPRS**							
Back	Mean ± SD	0.6 ± 1.7	2.4 ± 2.6	<0.001	0.4 ± 1.3	2.3 ± 2.7	<0.001
	*P* value (within group)	0.005	<0.001	–	0.012	<0.001	–
Leg†	Mean ± SD	0.9 ± 2.0	2.5 ± 3.0	<0.001	1.4 ± 2.1	3.6 ± 3.1	<0.001
	*P* value (within group)	<0.001	<0.001	–	<0.001	<0.001	–
**ZCQ**							
Symptom severity	Mean ± SD	0.11 ± 0.48	0.72 ± 0.85	<0.001	0.12 ± 0.46	0.64 ± 0.83	<0.001
	*P* value (within group)	0.096^§^	<0.001	–	0.026	<0.001	–
Physical function	Mean ± SD	0.05 ± 0.35	0.48 ± 0.65	<0.001	0.04 ± 0.38	0.43 ± 0.70	<0.001
	*P* value (within group)	0.199^§^	<0.001	–	0.412^§^	<0.001	–
Patient satisfaction^ǂ^	Mean ± SD	2.71 ± 0.90	2.19 ± 0.88	0.001	2.84 ± 0.89	2.27 ± 0.81	<0.001

* Two-tailed *t* test assuming unequal sample variances (independent samples).

† Pain with the lowest value used for analysis.

‡ No imputation for patients receiving a subsequent lumbar spine intervention, ZCQ Patient Satisfaction threshold ≤2.5.

§ Within-group mean change was not statistically significant.

Mean improvement from baseline to 1-year follow-up for all secondary outcome measures, including NPRS for back and leg and the ZCQ physical function and symptom severity domains, demonstrated statistical significance in favor of *mild*+CMM vs CMM-Alone (*P* < 0.001 for all outcome measures). ZCQ patient satisfaction also indicated that *mild*+CMM patients were statistically significantly more satisfied with their treatment than were CMM-Alone patients at 1-year follow-up (*P* < 0.001). For ZCQ patient satisfaction, lower scores indicate greater patient satisfaction with their treatment, and scores ≤2.5 indicate that the patient is satisfied [17]. A within-group analysis showed that mean improvements for all primary and secondary outcome measures were statistically significant for *mild*+CMM at both the 6-month and 1-year follow-ups. Within-group mean improvements for certain outcome measures for CMM-Alone patients did not reach statistical significance, including ODI and ZCQ physical function at 1-year follow-up and ZCQ symptom severity and physical function at 6-month follow-up. See Table 5 for comprehensive outcome data at 6-month and 1-year follow-up.

## Discussion

This prospective, multicenter, randomized controlled trial comparing *mild*+CMM with CMM-Alone provides Level-1 evidence that the *mild* Procedure together with CMM is superior to CMM alone in improving function and decreasing pain for patients with LSS suffering from NC and HLF. The *mild* Procedure removes a major root cause of LSS by debulking the thickened ligamentum flavum, preserves baseline spine stability, and leaves no implants behind. The *mild* Procedure has a safety profile similar to that of epidural steroid injections, and patients typically resume normal activity within 24 hours with no restrictions. Measurement of increased ability to walk without severe symptoms via the validated Walking Tolerance Test, incidence of subsequent lumbar spine interventions, and frequency of adverse events provide objective assessments of patient quality of recovery in the real-world clinical environment. The outcomes of this study reflect routine use of the *mild* Procedure in a typical clinic setting.

*mild*+CMM showed a statistically significant improvement over CMM-Alone and within group for the ODI composite primary endpoint and secondary NPRS and ZCQ endpoints, along with sustained functional improvement at 1 year. This sustained improvement from baseline to 1 year within *mild*+CMM, while maintaining a wide margin of difference between the groups, shows the durable impact of the *mild* Procedure together with adjunctive conservative therapies. This durability has been seen in many previous studies reporting on similar outcome measures at 1-, 2-, and 5-year follow-up [10, 24–29]. Clinically meaningful results have also been seen in walking and standing duration as a result of the *mild* treatment. One year after treatment with *mild*, Mekhail and colleagues at Cleveland Clinic reported a seven-fold improvement in standing time, from 8 minutes to 56 minutes, and a 16-fold increase in walking distance, from 246 feet to 3,956 feet [26]. The MOTION study supports these results with significant improvement from baseline to follow-up in the Walking Tolerance Test at 6 months (228% increase in walking time over baseline) and maintenance of this improvement at 1 year (258% increase). The MOTION study Walking Tolerance Test improvement was limited by capping the walking time to 15 minutes, whereas in the study by Mekhail et al., study patients walked until the onset of NC symptoms.

The incidence of subsequent lumbar spine interventions can be considered as a measure of patient dissatisfaction with the initial treatment and continued need for symptom relief. Although only 5.8% of patients in *mild*+CMM felt that they needed additional interventions beyond conservative therapies over the course of a year, 26.1% of patients in CMM-Alone sought subsequent lumbar spine interventions during the same time period, of which half were *mild* Procedures. The greater level of satisfaction with *mild*+CMM is reflected in the significantly better satisfaction scores for this treatment arm in the ZCQ questionnaire. The rate of crossover to subsequent lumbar spine interventions in the CMM-Alone group reinforces the position of *mild* as first-line therapy in real-world settings.

No device- or procedure-related adverse events or SAEs occurred in the MOTION study. This level of safety reflects the low rates of complications in previous studies comparing the *mild* Procedure with epidural steroid injection, including the ENCORE randomized controlled trial, in which there was no difference in the rate of adverse events between the two treatment groups (1.3%, *P* = 1.00) [9, 10, 30–32]. The safety of the *mild* Procedure has also been proved extensively in multiple studies that reported no major device- or procedure-related SAEs [24–28].

The *mild* Procedure is not intended to directly treat lateral or foraminal stenosis, but reports have indicated that related bony anomalies were actually a positive predictor of success with *mild* [33]. The MOTION study confirms the outcomes of previous studies, in which the *mild* Procedure has been shown to be safe and effective for patients with multiple comorbidities, such as foraminal narrowing, lateral recess stenosis, bulging disc, and facet arthropathy, in addition to HLF [10, 24, 30]. Nearly all MOTION patients (92.9%) presented with foraminal and central stenosis, and more than half suffered from all three types of stenosis (foraminal, lateral, and central). Furthermore, more than 90% presented with at least five spinal comorbidities. There was no statistical difference for any baseline characteristic or comorbidity between the two treatment groups, precluding potential bias in the results from these conditions and illustrating the impact that the *mild* Procedure can have on patients with typical constellations of related spinal comorbidities.

Although the MOTION study was designed to include a patient population commonly seen every day in the clinic, the inclusion of numerous CMM treatment options chosen at the investigator’s discretion provided a broad range of treatment options and sequencing, as is encountered in the real world. This limited control over the use of CMM, though intended in the study design, may be viewed as a study limitation. The use of CMM in both arms of this study simulates real-world practice, but it also may result in confounding, as patients are treated on the basis of routine use of the *mild* Procedure in a typical clinic setting. In day-to-day practice, the *mild* Procedure is not used alone but in conjunction with other conservative therapies. The nonblinded nature of the study can also be considered a limitation. The use of objective real-world outcome measures, together with independent physicians in the role of medical monitor, clinical events adjudicator, and study principal investigator, were intended to limit study bias.

## Conclusion

The 1-year results of the Level-1 MOTION randomized controlled trial demonstrate the superiority of the *mild* Procedure in combination with CMM over the use of CMM alone for patients with LSS suffering from NC secondary to HLF. The *mild* Procedure with CMM was shown to be durable to 1 year through validated, patient-reported outcomes and objective real-world measures. Use of the validated Walking Tolerance Test to objectively measure increased ability to walk without severe symptoms provided evidence of statistically significantly better outcomes for *mild*+CMM than for CMM-Alone. With no reported device- or procedure-related adverse events, the long-standing safety profile of the *mild* Procedure was validated. The results of this study confirm the use of the *mild* Procedure as a safe and effective first-line treatment for the indicated LSS patient population.

## Notes

Funding source: This study is sponsored by Vertos Medical, Inc.

Disclosure and conflicts of interest: Timothy Deer is the research principal investigator on this study and is a consultant for Abbott, Saluda, CornerLoc, PainTEQ, Nalu, Boston Scientific (Vertiflex), Vertos, and Medtronic (Stimgenics). He has received research funding from Abbott, Boston Scientific, Vertos, and Avanos. Shrif Costandi has received research funding from Vertos. Edward Washabaugh has received research funding from Boston Scientific and Vertos. Tim Chafin has received research funding from Vertos. Sayed Wahezi is a consultant for Boston Scientific and has received research funding from Vertos. Navdeep Jassal is a consultant for Vertos, Abbott, Boston Scientific, Flowonix, CornerLoc, and PainTEQ and has received research funding from Vertos, PainTEQ, and Flowonix. Dawood Sayed is a consultant for Abbott, Medtronic, Nevro, SPR, Vertos, and PainTEQ and has received research funding from and holds minority equity in Vertos. The authors report no other conflicts of interest in relation to this work.

**Trial registration:** ClinicalTrials.gov registration number NCT03610737.
